# Filling the treatment gaps in osteoporosis: osteoimmunology as a new frontier

**DOI:** 10.1016/j.ebiom.2026.106221

**Published:** 2026-03-11

**Authors:** eBioMedicine

The December, 2025, approval by the US Food and Drug Administration (FDA) of hip bone mineral density as a surrogate endpoint for osteoporosis drug development marks a new era in the management of this condition, paving the way for faster and more efficient development of treatments, and providing a great alternative to fracture endpoints. Instead of waiting for endpoints such as fractures, these surrogate endpoints could substantially shorten the lifecycle of drug approval, accelerating access to new therapies. This regulatory change could have notable implications for addressing the effect of osteoporosis on the 500 million people worldwide with this condition.

The need for new and efficient treatments in osteoporosis remains high. Osteoimmunology, an emerging interdisciplinary area that explores the interplay between the bone and immune system, appears promising for developing drugs that help regulate the immune microenvironment of the bone. Currently, denosumab is the only approved drug for osteoporosis that directly targets immunological pathways. Denosumab is a human monoclonal antibody that inhibits RANKL, a key factor in promoting osteoclast formation, thus decreasing bone resorption and increasing bone mass and strength. The success of denosumab strongly puts the immune system into the frame for future osteoporosis treatments.

Beyond RANKL, other cytokines have emerged as drivers of osteoclast activation and inflammatory bone loss, making them ideal targets for therapeutic approaches. IL-17, IL6, and TNF have been shown to mediate osteoclastogenesis and inflammatory bone loss, contributing to the dysregulation of bone remodelling in osteoporosis, and they have shown strong translational potential as emerging cytokine targets in osteoimmunology. Studies have also explored new mechanistic insights and immune pathways that might shape the bone immune microenvironment. For example, a recent study in rats, published in *eBioMedicine*, unveiled a signalling pathway between the muscle and bones whereby cytokines secreted by skeletal muscle play a role in osteogenesis. Although these findings remain at a preclinical stage, they highlight promising avenues for future drug development and the importance of muscle health in the condition.

Immune cells also play an important role in shaping the bone immune microenvironment. Regulatory T cells and macrophages in in vitro studies and animal models have shown the benefits of regulating the immune microenvironment through the modulation of inflammatory cells. Preclinical studies suggest that regulatory T cell-derived exosomes can enhance mesenchymal stem cell-mediated repair processes, thereby accelerating fracture repair. Wharton's jelly-derived mesenchymal stem cells, which among other immunomodulatory properties have been shown to induce regulatory T cells, are currently being used in an ongoing clinical trial in combination with the drug teriparatide for osteoporotic vertebral fractures.

Aside from established approaches, novel avenues for modulating the immune system are emerging, one being biomaterial-based immunomodulation. A recently published review focused on manganese-based nanozymes, nanoparticles that combine the beneficial properties of nanomaterials, manganese-specific characteristics, and multi-enzyme mimetic activities, capable of remodelling the immune microenvironment. Although not yet clinically applied in osteoporosis, immunomodulatory nanomedicine has shown potential in bone tissue engineering. Of course, the implementation of nanomaterials in therapies is a substantial undertaking that comes with its own set of limitations, including toxicity and immunogenicity.

In addition to immune-cell and biomaterial driven strategies, the gut microbiome is an emerging player in shaping immune status to prevent bone loss through the action of short chain fatty acids, which supress pro-inflammatory pathways that drive osteoclast formation. A study in mice with osteoporosis showed that faecal microbiota transplantation from donor mice of the same age without ovariectomy inhibited bone loss and suppressed the release of pro-osteoclastogenic cytokines. Probiotics such as *Clostridium butyricum* have been shown to reduce radiation-induced bone loss in mice and might form promising and relatively simple approaches. Such microbiome-based interventions, which also include the use of prebiotics and high-fibre diets, could offer accessible treatment possibilities.

Taken together, the above bring into the spotlight the potential of immunomodulatory treatments in osteoporosis. The successful use of denosumab has paved the way for further research into immunological targets, and there is a plethora of immune-bone signalling pathways to explore further. The complexity of the immunological mechanisms underlying osteoporosis and the lack of immune biomarkers as endpoints are two of the main challenges that need to be addressed. Multi-omics approaches for immune profiling might prove instrumental in identifying promising new immunological targets for osteoporosis and enabling the development of predictive and prognostic biomarkers for osteoporosis.

Even though ongoing investigations are mostly at pre-clinical stages, the proven success of immunomodulatory therapies in cancer and autoimmune disorders makes osteoimmunology a ripe ground for research. *eBioMedicine* remains committed to supporting high-quality translational research and encourages submissions that help advance knowledge in osteoimmunology and enable the development of new treatments for osteoporosis.

